# Analysis of the Relationship Between EGFR Mutations and PD-L1, ALK, and ROS1 Alterations in Patients with Non-Small-Cell Lung Cancer: The Most Extensive Study Conducted in Iran

**DOI:** 10.5146/tjpath.2025.13827

**Published:** 2025-09-30

**Authors:** Sepideh Hadimaleki, Roham Sarmadian, Abolfazl Gilani, Parisa Mehrasa, Ali Esfahani, Mortaza Raeisi, Yousef Roosta, Amir Vahedi

**Affiliations:** Department of Pathology, Tabriz University of Medical Sciences, Tabriz, Iran; Infectious Disease Research Center, Arak University of Medical Sciences, Arak, Iran; Department of Pathology, Tehran University of Medical Sciences, Tehran, Iran; Department of Oncology, Tabriz University of Medical Sciences, Tabriz, Iran; Department of Hematology and Oncology Research Center, Tabriz University of Medical Sciences, Tabriz, Iran

**Keywords:** Non-small-cell lung cancer, EGFR mutation, PD-L1 expression, ALK rearrangement, ROS1 rearrangement

## Abstract

***Objective: *** Lung cancer, the second most common type of cancer, is the leading cause of cancer-related mortality, with non-small-cell lung carcinoma (NSCLC) being the most prevalent subtype. The presence of *EGFR* mutations in NSCLC influences tumor behavior and treatment response. The prevalence of *EGFR* mutation in Iranian patients is limited. This study investigated the frequency of *EGFR* mutation and its association with PD-L1, *ALK*, and *ROS1* expression in patients with NSCLC from Northwest Iran.

***Material and Methods:*** A retrospective analysis was conducted on 647 cases of NSCLC from April 2018 to August 2024 at Imam Reza Hospital in Tabriz, Iran. Histologic diagnoses were confirmed, and patient data were collected. *EGFR* mutation testing targeted exons 18-21 using Sanger sequencing and Real-Time PCR. *ALK* and *ROS1* rearrangements were assessed using fluorescence in situ hybridization (FISH), while PD-L1 expression was evaluated through immunohistochemistry (IHC). The statistical analysis was performed using SPSS version 27.0.

***Results: *** The cohort comprised 430 males and 217 females, with a median age of 62 years (IQR: 54-70). *EGFR* mutations were identified in 171 (26.4%) cases, more frequently in females (33.6% vs. 22.8%; p = 0.003). The most common mutation was exon 19 deletion (56.7%), followed by L858R (21.6%). No significant association was found between *EGFR* mutations and *ALK* (p = 0.126) or PD-L1 expressions (p = 0.29). *ROS1* mutations were not detected.

***Conclusion:*** This study confirmed the mutual exclusivity of *EGFR* and *ALK* mutations and found no significant association with PD-L1. Comprehensive *EGFR* testing remains crucial to guide targeted therapies. Broader studies are needed to include diverse populations and additional clinical factors to improve personalized treatment.

## INTRODUCTION

Lung cancer is the second most common type of cancer and the primary cause of cancer-related fatalities in both men and women (1). It is estimated that lung cancer deaths could reach nearly 3 million by 2035 (2). Generally, lung malignancies are categorized into small-cell lung carcinoma (SCLC) and non-small-cell lung carcinoma (NSCLC), with NSCLC being the most frequently diagnosed type, accounting for over 50% of cases. In NSCLC, the signaling pathways involving the ErbB protein family become dysregulated, leading to increased malignant cell growth, resistance to programmed cell death, and enhanced migration and metastasis (3). The epidermal growth factor receptor (*EGFR*) is a 170-kilodalton transmembrane protein that belongs to the ErbB family of tyrosine kinases located on chromosome 7. When *EGFR* is dysregulated, it boosts the activity of intracellular pathways through tyrosine kinase autophosphorylation, resulting in increased DNA synthesis, cell proliferation, invasion, and metastasis (4).

Previous research indicates that *EGFR* is overexpressed in approximately 10%–41% of lung adenocarcinomas and highlights specific activating mutations in the *EGFR* gene, particularly in exons 18, 19, 20, and 21, which lead to sensitivity to tyrosine kinase inhibitors (TKIs), such as gefitinib (5). The prevalence of *EGFR* mutations in non-small-cell lung cancer (NSCLC) varies significantly among different ethnic groups, influencing patient responses to *EGFR*-targeted therapies. For example, East Asian populations exhibit the highest rates of *EGFR* mutations, estimated at 30%–50%, which are predominantly responsive to TKIs. In contrast, many African American populations have a considerably lower incidence of *EGFR* mutations, with most studies suggesting rates of approximately 5%–10%. Nonetheless, recent studies have indicated that African Americans with *EGFR* mutations can still benefit from TKI therapy (6,7). NSCLC represents a considerable public health challenge in Iran, and it is characterized by a high prevalence of adenocarcinoma and significant rates of *EGFR* mutations (8).

However, few investigations have been conducted on *EGFR* gene mutations in Iranian patients with NSCLC. Therefore, the current study aimed to evaluate *EGFR* mutation frequencies and the association between the rearrangement rates of PD-L1 expression, *ALK*, and *ROS1* with *EGFR* mutations in 647 patients with NSCLC at the largest referral center in Northwest Iran.

## MATERIAL and METHODS

This 6-year retrospective study (April 2018 to August 2024) included 647 patients with confirmed NSCLC. Among them, 171 patients had an *EGFR* mutation. Data were obtained from patients referred to Imam Reza Hospital in Tabriz, Iran. Demographic characteristics were recorded using the Hospital Information System (HIS). Hematoxylin and eosin (H&E) slides were evaluated by two board-certified pathologists to confirm the histologic diagnosis of NSCLC. Mutation analysis was conducted using formalin-fixed paraffin-embedded (FFPE) tissues obtained from primary or metastatic tumor sites. Genomic DNA was isolated from the patient using a Qiagen (USA) DNA extraction kit. Following DNA extraction, PCR amplification was performed to target exons 18–21 of the *EGFR* gene. PCR products were then sequenced using the Sanger method. To improve the specificity and sensitivity of the study, PCR of these exons was conducted in both the sense and antisense directions, and the primary PCR product was used in nested PCR. Although we performed real-time PCR with the Diatech Kit for the evaluation of seven mutations (G719x, T790M, S768I, ex20ins, L858R, L861Q, and ex19del), all results, conventional and Real-time, were analyzed and calculated. To comprehensively evaluate key genetic and protein biomarkers in non-small cell lung carcinoma (NSCLC), fluorescence in situ hybridization (FISH) and immunohistochemistry (IHC) analyses were employed to assess the status of *ALK*, *ROS1*, and PD-L1 in tumor samples.

For detecting the *ALK*-*EML4* fusion gene, FISH analysis was conducted using the Cytocell *ALK* break-apart probe kit, which targets rearrangements in the *ALK* gene on chromosome 2p23. Following the manufacturer’s protocol, samples were prepared, and probe hybridization was carried out, followed by washing to ensure signal clarity. Cells displaying split red and green signals under a fluorescence microscope were marked as positive for *ALK* rearrangement, suggesting potential responsiveness to *ALK*-targeted therapies. Similarly, to evaluate *ROS1* gene rearrangements, FISH analysis was performed using the Diagen *ROS1* break-apart probe kit. This assay identifies rearrangements in the *ROS1* gene on chromosome 6q22, which may indicate eligibility for *ROS1*-specific inhibitors. The procedure involved sample preparation, probe hybridization, and subsequent washing, as recommended in the Diagen protocol. Fluorescent signals were analyzed microscopically, with cells showing split red and green signals classified as *ROS1*-positive. To assess PD-L1 expression, immunohistochemistry (IHC) was performed using the PD-L1 IHC 22C3 pharmDx kit from Master Diagnostics. This assay employs a monoclonal antibody specific for PD-L1 (clone 22C3) and is validated for use on formalin-fixed, paraffin-embedded (FFPE) tissue sections. The IHC protocol included antigen retrieval, primary antibody incubation, and detection with a horseradish peroxidase (HRP) system, and the percentage of positive tumor cells were evaluated under light microscopy, with PD-L1 levels reported as the tumor proportion score (TPS) to indicate the extent of PD-L1 expression in tumor samples.

Descriptive statistics were used to summarize the demographic and clinical characteristics of the study population. Continuous variables were presented as the median and interquartile range (IQR) since the data were not normally distributed, as confirmed by the Kolmogorov-Smirnov test. Categorical variables were expressed as frequencies and percentages. For comparisons between groups, the following statistical tests were applied: Mann-Whitney U test, Fisher’s exact test. A p-value < 0.05 was considered statistically significant. All statistical analyses were performed using SPSS version 27.0. This study adhered to the Helsinki Declaration and was approved by the Ethics Committee of Tabriz University of Medical Sciences, Tabriz, Iran (Reference number: IR.TBZMED.REC.1398.091).

## RESULTS

Our study included 647 cases of NSCLC. Of these, 430 cases were males (66.5%) and 217 cases were females (33.5%). The median age was 62 years (IQR: 54-70). Among the 647 specimens, 633 specimens (97.8%) were obtained from biopsies and 14 (2.2%) specimens were resected surgically. The overall frequencies of the *EGFR*, *ALK*, *ROS1* alterations are summarized in Table 1.

**Table 1:** The alteration types and frequencies in our cases

**Gene**	**Frequency**
*EGFR* mutation	26.4% (171 of 647)
*ALK *rearrangement	2.3% (15 of 647)
*ROS1 *rearrangement	0%
PD-L1 expression	54.8% (355 of 647)

**Figure 1:**
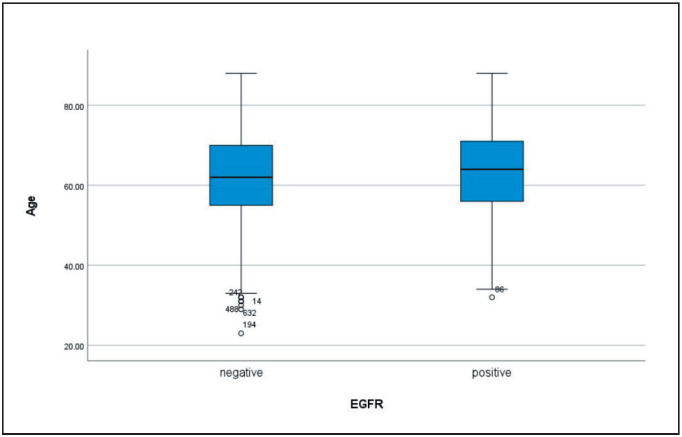
Box plot chart of age in two groups (EGFR positive and negative).

Among the 171 studied cases (26.4%) with *EGFR* mutation, 165 cases (96.5%) were primary, and 6 (3.5%) cases were metastatic. Our results showed that *EGFR* mutations were more frequent in women than in men (33.6% versus 22.8%) (p=0.003). Also, the median age of *EGFR*-positive cases was 63 (IQR: 55-70), compared to 61 years (IQR: 53-69) in the *EGFR*-negative group (Mann-Whitney U test, p = 0.15) (Figure 1). The most common *EGFR* mutation was exon 19 deletion (56.7%), followed by L858R point mutation (21.6%) and exon 20 insertion (4.1%). Other mutations, including G719X, S768I, and L861Q, were observed in smaller proportions (Table 2). *ALK* rearrangements were detected in 15 cases (2.3%), with no significant association between *EGFR* mutations and *ALK* rearrangements (Fisher’s exact test, p = 0.126). The mutual exclusivity of *EGFR* and *ALK* mutations was consistent with findings from previous studies. No *ROS1* rearrangements were identified in the cohort. PD-L1 expression was categorized as (TPS < 1%: 45.2% of cases, TPS 1–49%: 32.1% of cases, TPS ≥ 50%: 22.7% of cases). There was no significant association between PD-L1 expression and *EGFR* mutation status (Fisher’s exact test, p = 0.29) (Table 3, Table 4).

**Table 2:** The EGFR mutation subtypes and frequencies in the cases

**Mutation Subtype**	**Frequency**
Ex19Del	56.7%
L858R	21.6%
Ex20ins	4.1%
S768I	2.3%
T790M	4.7%
L861Q	6.4%
G719X	1.8%
G787A	1.8%
G719C	0.6%

**Table 3:** Results of ALK, ROS1 and PD-L1 analyses

**Mutation**	**Status**	**Count**	**Percentage within EGFR**	**p-value**
*ALK*	Negative	632	97.7%	0.126
	Positive	15	2.3%	
PD-L1	Negative	292	45.2%	0.29
	1%-49%	208	32.1%	
	50%<	147	22.7%	
*ROS1*	Negative	647	100%	-
	Positive	0	0%	

**Table 4:** Demographic and Molecular Characteristics

**Variable**	**Total (n = 647)**	**EGFR+ (n = 171)**	**EGFR- (n = 476)**	**p-value**
Age	62 (54-70)	63 (55-70)	61 (53-69)	0.15*
Gender				0.003**
Male	430 (66.5%)	98 (22.8%)	332 (77.2%)	
Female	217 (33.5%)	73 (33.6%)	144 (66.4%)	
*ALK* rearrangement	15 (2.3%)	5 (2.9%)	10 (2.1%)	0.126**
PD-L1 Expression				0.29**
TPS<1%	292 (45.2%)	75 (43.9%)	217 (45.6%)	
TPS:1%-49%	208 (32.1%)	55 (32.2%)	153 (32.1%)	
TPS>50%	147 (22.7%)	41 (24%)	106 (22.3%)	

*Mann-Whitney U test, **Fisher’s exact test

While specific histopathological subtypes (e.g., adenocarcinoma, squamous cell carcinoma) were not detailed in the dataset, the high prevalence of *EGFR* mutations suggests a predominance of adenocarcinoma, as *EGFR* mutations are more commonly associated with this subtype. Further studies with detailed histopathological data are needed to confirm this finding.

## DISCUSSION

*EGFR* mutations play a pivotal role in the clinical management of NSCLC, influencing both patient demographics and therapeutic responses. As one of the most common genetic alterations in NSCLC, particularly in adenocarcinoma subtypes, *EGFR* mutations have been extensively studied to understand their implications for targeted therapies. The presence of these mutations is associated with favorable outcomes when treated with *EGFR* tyrosine kinase inhibitors (TKIs), which have revolutionized the treatment landscape for NSCLC (9). However, the relationship between *EGFR* mutation status and patient characteristics, such as age, sex, and other gene expressions, is complex and warrants further exploration. This study aimed to elucidate the demographic and molecular characteristics of patients with NSCLC and *EGFR* mutations in a large Iranian cohort. Patients with *EGFR* mutations in NSCLC exhibit distinct clinical and demographic characteristics influencing treatment responses. Our findings confirm that *EGFR* mutations are more prevalent in females (33.6%) than in males (22.8%, p=0.003), consistent with previous studies (10). The higher prevalence of *EGFR* mutations in females aligns with global trends, particularly in East Asian populations, where *EGFR* mutations are found in 30-50% of NSCLC cases (11). Additionally, the mean age of *EGFR*-positive patients (63 years) was slightly higher than that of *EGFR*-negative patients (61 years), although this difference was not statistically significant. This finding contrasts with some studies in which younger age was associated with a higher likelihood of certain mutations but lower overall rates of *EGFR* mutations. For instance, a study found that among female NSCLC patients, those aged over 57 years had a higher prevalence of *EGFR* mutations (70%) compared with younger patients (39%) (12), highlighting the need for further investigation into age-related differences in *EGFR* mutation prevalence.

This gender disparity may be attributed to hormonal influences or differences in smoking patterns, as *EGFR* mutations are more common in never-smokers (12). Although smoking status was not available in our dataset, some studies show that never-smokers have a higher prevalence of *EGFR* mutations than smokers. For example, in a meta-analysis, the mutation rates were reported at 70% for never-smokers versus 41.9% for smokers, indicating a strong inverse relationship between smoking and the *EGFR* mutation status (11). In a study by Parvar et al., 81.8% of the patients with *EGFR* mutations were never-smokers, highlighting the association between nonsmoking status and positive *EGFR* mutations (13). Moreover, current smokers with *EGFR* mutations often have worse overall survival compared with non-smokers (14).

Histologically, most *EGFR*-mutant NSCLC cases are adenocarcinomas, which is the most common lung cancer subtype among non-smokers (15). In East Asian populations, the prevalence of *EGFR* mutations is 47.9% in adenocarcinoma and 4.6% in lung squamous cell carcinoma, while in Western people, it is 19.2% in lung adenocarcinoma and 3.3% in lung squamous cell carcinoma (16). The most frequent *EGFR* mutations include exon 19 deletions, occurring in 50-60% of cases, and the L858R point mutation in exon 21, present in about 30% of cases. Less common mutations, such as exon 20 insertions, are also observed at lower frequencies (17). Our study similarly revealed that the most prevalent mutations were exon 19 deletion (56.7%), L858R point mutation (21.6%), and exon 20 insertion (4.1%), consistent with global data. Patients with positive *EGFR* mutation generally respond favorably to *EGFR* tyrosine kinase inhibitors (TKIs) such as gefitinib and osimertinib (18,19). Studies have shown that progression-free survival (PFS) is usually longer in patients receiving TKIs compared with those receiving standard chemotherapy, often exceeding 10 months (20). However, the presence of less common mutations, such as exon 20 insertions, underscores the importance of comprehensive molecular testing to guide personalized treatment strategies. In our study, no significant association was observed between PD-L1 expression and *EGFR* mutation status (p = 0.29). This finding contrasts with some studies that reported higher PD-L1 expression in *EGFR*-mutant tumors (21,22), while others suggest a negative correlation (23). The lack of consistency in these results may reflect differences in study populations, methodologies, or ethnic gene expression. For instance, Tang et al. reported PD-L1 overexpression in 71.9% of *EGFR*-mutant NSCLC cases, compared to 57.1% in wild-type cases (22). Meanwhile, Kojima et al. found that *EGFR* mutations were associated with lower PD-L1 expression and poorer responses to immune checkpoint inhibitors (24). These discrepancies highlight the need for further research to clarify the relationship between *EGFR* mutations and PD-L1 expression, particularly in diverse populations. Studies have shown that *ROS1* rearrangements and *EGFR* mutations are generally mutually exclusive events in NSCLC (25). For instance, a comprehensive review noted that while *ROS1* rearrangements occur in approximately 1-2% of NSCLC cases, concurrent occurrences with *EGFR* mutations are extremely rare, with only a few documented cases in the literature (26). Prognostically, both mutations improve responses to targeted therapies (21,25). Similarly, in the present study, no *ROS1* Rearrangements were detected. Our findings support the mutual exclusivity of *EGFR* mutations and *ALK* rearrangements, as only 2.9% of *EGFR*-positive cases harbored concurrent *ALK* rearrangements (p = 0.126). This is consistent with previous studies. For example, in a study by Yang et al., out of 2975 EGFR-positive patients, only nine had concurrent mutations involving both genes (27). The rarity of cooccurrence implies that testing for one mutation often leads to the exclusion of testing for the other unless specific clinical characteristics suggest otherwise.

One of the key strengths of this study is its large sample size (n = 647), which is the largest cohort of patients with NSCLC and *EGFR* mutations studied in Iran to date. This study provides valuable insights into the demographic and molecular characteristics of *EGFR*-mutant NSCLC in an understudied population. However, several limitations should be acknowledged. First, the lack of data on smoking status, tumor stage, and histopathological subtypes limits our exploration of the relationship between these factors and *EGFR* mutation status. Second, the retrospective nature of the study may have introduced selection bias. Finally, the absence of survival data precludes an analysis of the impact of *EGFR* mutations on patient outcomes.

## CONCLUSION

This study contributes to the growing body of literature on *EGFR* mutations in NSCLC by providing data from a large Iranian cohort. Our findings confirm that the *EGFR* mutations in NSCLC are associated with distinct demographic characteristics such as female sex and older age. The high prevalence of common mutations like exon 19 deletions and L858R underscores the utility of *EGFR*-TKIs, which generally improve progression-free survival compared with chemotherapy. The lack of a significant association between *EGFR* mutations and PD-L1 expression underscores the need for further research to clarify the role of immune checkpoint inhibitors in *EGFR*-mutant NSCLC. Additionally, the mutual exclusivity of *EGFR* mutations and *ALK*/*ROS1* rearrangements reinforces the importance of sequential molecular testing in NSCLC management. Future studies should aim to include a more diverse patient population, encompassing various ethnicities and geographic regions, to better understand the global prevalence and characteristics of *EGFR* mutations in NSCLC. Additionally, incorporating data on smoking status, tumor stage, and histopathological subtypes would provide a more comprehensive understanding of the factors influencing *EGFR* mutation status and treatment outcomes. Finally, prospective studies using survival data are needed to evaluate the long-term impact of *EGFR* mutations on patient outcomes and to optimize personalized treatment strategies.

## Conflict of Interest

The authors have no conflicts of interest to declare.
